# A statewide review of postnatal care in private hospitals in Victoria, Australia

**DOI:** 10.1186/1471-2393-10-26

**Published:** 2010-05-28

**Authors:** Jo-Anne Rayner, Helen L McLachlan, Della A Forster, Louise Peters, Jane Yelland

**Affiliations:** 1 Mother and Child Health Research, La Trobe University, 324-328 Little Lonsdale St, Melbourne Victoria, 3000, Australia; 2 Division of Nursing and Midwifery, La Trobe University, Bundoora Victoria, 3086, Australia; 3 The Women's Hospital, Crn Flemington Rd and Grattan St, Parkville Victoria, 3052, Australia; 4 Healthy Mothers Healthy Families Research Group, Murdoch Childrens Research Institute, PO Box 911, Parkville Victoria, 3052, Australia

## Abstract

**Background:**

Concerns have been raised in Australia and internationally regarding the quality and effectiveness of hospital postnatal care, although Australian women receiving postnatal care in the private maternity sector rate their satisfaction with care more highly than women receiving public maternity care. In Victoria, Australia, two-thirds of women receive their maternity care in the public sector and the remainder in private health care sector. A statewide review of public hospital postnatal care in Victoria from the perspective of care providers found many barriers to care provision including the busyness of postnatal wards, inadequate staffing and priority being given to other episodes of care; however the study did not include private hospitals. The aim of this study was replicate the review in the private sector, to explore the structure and organisation of postnatal care in private hospitals and identify those aspects of care potentially impacting on women's experiences and maternal and infant care. This provides a more complete overview of the organisational structures and processes in postnatal care in all Victorian hospitals from the perspective of care providers.

**Methods:**

A mixed method design was used. A structured postal survey was sent to all Victorian private hospitals (n = 19) and key informant interviews were undertaken with selected clinical midwives, maternity unit managers and obstetricians (n = 11). Survey data were analysed using descriptive statistics and interview data analysed thematically.

**Results:**

Private hospital care providers report that postnatal care is provided in very busy environments, and that meeting the aims of postnatal care (breastfeeding support, education of parents and facilitating rest and recovery for women following birth) was difficult in the context of increased acuity of postnatal care; prioritising of other areas over postnatal care; high midwife-to-woman ratios; and the number and frequency of visitors. These findings were similar to the public review. Organisational differences in postnatal care were found between the two sectors: private hospitals are more likely to have a separate postnatal care unit with single rooms and can accommodate partners' over-night; very few have a policy of infant rooming-in; and most have well-baby nurseries. Private hospitals are also more likely to employ staff other than midwives, have fewer core postnatal staff and have a greater dependence on casual and bank staff to provide postnatal care.

**Conclusions:**

There are similarities and differences in the organisation and provision of private postnatal care compared to postnatal care in public hospitals. Key differences between the two sectors relate to the organisational and aesthetic aspects of service provision rather than the delivery of postnatal care. The key messages emerging from both reviews is the need to review and monitor the adequacy of staffing levels and to develop alternative approaches to postnatal care to improve this episode of care for women and care providers alike. We also recommend further research to provide a greater evidence-base for postnatal care provision.

## Background

Concerns have been raised in Australia and internationally regarding the quality and effectiveness of hospital postnatal care[1-4]. Women have consistently rated their postnatal care less favourably than other episodes of maternity care, leading to recommendations that postnatal care could be improved[1,3,5-7]. However, in two statewide surveys of recent mothers in Victoria, Australia, women cared for in the private maternity sector were significantly more likely to report higher satisfaction with their postnatal care than women in the public sector[3,5]. In the most recent survey, factors associated with higher ratings of satisfaction with postnatal care in both sectors were having a longer length of hospital stay, continuity of care, and the quality of women's experiences with care providers[3].

The provision of hospital services in Australia operates across two sectors - public and private, a system that has operated in Australia since the early twentieth century[8]. In private hospitals fees for accommodation and other services are charged directly to the consumer, which are reimbursed in most instances through private health insurance. In 2006, privately owned and operated hospitals represented 43% of all Australian hospitals,[9] and half the population over 15 years had private health insurance[10]. Consumers of private health care report peace of mind, shorter waiting periods, and the ability to choose one's own doctor as reasons for having insurance[10]. In 2000, the Australian national government introduced legislation to encourage the uptake of private health insurance, which created a financial loading additional to premiums that incrementally increase with age[11]. Since this time the numbers of beds and patients stays in private hospitals has continued to rise and new private hospitals have commenced operating, however there has been a decrease (1.8%) in the total number of staff employed in this sector[12].

Victoria is the smallest mainland state in Australia but has the second largest population - over five million people, the majority of whom reside in metropolitan areas. In Victoria women can choose publicly funded maternity care or private maternity care. Private maternity care is usually covered by private health insurance and most women have a private obstetrician as their lead care provider and give birth in a private hospital. In 2006, 30% (20,495/69,550) of women giving birth in Victoria gave birth in a private hospital, with two-thirds (64.2%; 47,551/69,550) giving birth in a public hospital, usually as a public patient. A small proportion of women have private maternity care but give birth in a public hospital (5.5%; 3,755/69,550)[13]. Women choosing private maternity care in Australia are generally older, more educated, non-smokers, and healthier;[14] and they experience fewer pregnancy complications[15,16] than women receiving public maternity care. However, they are more likely than women in the public sector to have obstetric interventions such as epidurals, induction of labour, instrumental and operative birth[16-18]. They also have longer lengths of postnatal hospital stay[13].

In 2004, the first statewide review of the organisation and provision of postnatal care in Victorian public hospitals from the perspective of care providers (PinC: Postnatal in hospital Care) was undertaken. The review involved a survey of all eligible public hospitals providing maternity services (n = 69) and interviews with key informants from selected hospitals (n = 38), including clinical midwives, managers of maternity units and medical practitioners. Sixty-six (96%) hospitals participated and the findings demonstrated diversity in the organisation of postnatal care across the state,[19] especially related to staffing arrangements[20] and 'routine' postnatal practices[19,21]. Barriers to care provision included the busyness of postnatal wards, the inadequacy of midwife-to-woman ratios, and the priority given to other episodes of care[19,20,22]. Similar findings have been reported elsewhere[4,23-26].

We were unable to identify any published literature nationally or internationally, on the provision of postnatal care in private hospitals. Given that one third of women in Victoria receive postnatal care in a private hospital we undertook a similar statewide review of private postnatal care (PinC Private). The aim of the study was to explore care providers' views of postnatal care provision in private hospitals in Victoria and to explore the similarities and differences in the provision of private and public postnatal care. This paper reports on the findings of this review and with the findings from the public review provides a more complete picture of the organisation and provision of postnatal hospital care in Victoria from the perspective of care providers.

## Methods

The review included a survey of all Victorian private maternity hospitals to ascertain the current organisation and provision of postnatal care, as well as interviews with key informants (KI) to explore care providers' experiences in depth. Research ethics approval was obtained from the Victorian Department of Human Services and La Trobe University Research Ethics Committees. No private provider required individual ethics committee review.

### Survey of private hospitals offering maternity services

The postal survey comprised open and closed-ended questions on a range of areas, based on the original PinC public review, which aimed to explore the structure and organisation of postnatal care in public hospitals and identify those aspects of care potentially impacting on women's experiences and maternal and infant care[19]. The original survey was developed by the research team, incorporating evidence from the literature and suggestions from the reference group established for the public review. The reference group brought together a range of people with relevant backgrounds and experience in postnatal care to contribute ideas and advice to the research team at all stages. Piloting of the survey was undertaken and a number of modifications and additions were made. Additional questions were incorporated in the private survey following feedback from a similar reference group established for the private review (Additional File 1). The questions related to practices that either happened only in the private sector (e.g. transfer to hotel care), or where it was considered that practices were different from the public sector (e.g. the provision of well-baby nurseries) (Additional File 2). Hospitals were asked about the organisation and provision of postnatal care and asked to send any documentation used in the provision of care[19].

Nineteen private hospitals were identified as providing in-hospital postnatal care in Victoria in 2006: 14 metropolitan, three regional and two rural. All 19 hospitals were invited to participate and the survey sent to either the Maternity Unit Manager or the Director of Nursing (in smaller services). After three weeks a courtesy call was made to those hospitals who had not returned the survey and two weeks after this a reminder letter was sent. No further contact was made with the hospitals following this. The survey data were entered onto an Access database[27] and analysed using STATA[28]. Hospitals were classified as either metropolitan or regional for analysis (regional and rural hospitals were combined). Pre-coded response questions were analysed using descriptive statistics. Open-ended responses were analysed using a thematic network[29]. This involves systematic extraction of basic codes from the data, which are grouped into organising categories and then inductively analysed into global themes. The systematic extraction of data creates a thematic network which illustrates the relationship between the organising themes and global themes.

Tables present data from the current study as well as from the original PinC Public review for comparison, to enable a ready visual comparison, as well as to facilitate the discussion later in the paper.

### Key informant interviews

Two sampling strategies were used to select key informants. Hospitals selected for interviews had an extra page added to their postal survey requesting contact details of three care providers who had agreed to be contacted by the research team to participate in an interview: a maternity unit manager or an associate unit manager; a clinical midwife; and an obstetrician. Initially five private hospitals (three metropolitan and two regional) were randomly selected and three agreed to participate in the key informant interviews. One did not nominate any care providers for interview and another did not respond to the postal survey therefore another hospital was purposively selected because of its postnatal care practice of early discharge to hotel-based care. Key informant interviews were chosen as they provide rich data from the individual perspective and allow for clarification and discussion of emerging issues. Interviewing a range of care providers enabled differing perspectives and experiences of postnatal care to be heard.

Interviews were conducted face-to-face or by telephone by members of the research team (LP, JR or HMc) and digitally recorded. Written consent was obtained from key-informants prior to commencement of each interview. Interview questions explored care providers' views on the structure and organisation of postnatal care, aims of postnatal care and women's expectations of hospital postnatal care in the private sector.

Interviews were transcribed verbatim using word processing software, coded thematically in the same manner as the open-ended survey questions[29] and cross-checked for inter-coder reliability. The analysis of the interviews identified two global themes describing the key informants views of private postnatal care: *constrained care *related to barriers impeding postnatal care such as the busy postnatal environment, staffing difficulties and visitors; and *consumer care *which describes care providers' views that increasingly women approached postnatal care as health consumers and maternity units were organised to accommodate this approach. These findings are reported in detail elsewhere[30] but relevant data from the interviews are included in this paper.

To maintain confidentiality and anonymity each participating hospital and key informant were allocated a unique identifier (ID). Quotes are used in this paper to illustrate findings and are identified by data source (survey or key informant interview), informant designation, hospital category, and ID.

## Results

### Response

Postal surveys were sent to all 19 private maternity service providers in June 2006. Ten of the 14 metropolitan and four of the five regional private hospitals completed and returned the survey, a response fraction of 74%. Eleven interviews were undertaken with key informants from three metropolitan and one regional private hospital in October 2006. Key informants included two maternity unit managers, six clinical midwives, and three obstetricians. Nine interviews were conducted face-to-face, with two undertaken by telephone at the request of the key informant. All key informants had considerable experience in postnatal care provision and had worked in maternity services for over ten years.

Participating hospitals were asked to provide 2005 patient profile data collected by the Victorian Perinatal Data Collection Unit (PDCU). The data showed that the majority of women who receive postnatal care in the participating private hospitals were: English-speaking (median 94%); over 30 years of age (median 81% in metropolitan, 73% in regional private hospitals); and Australian born (84%) (Table 1). The number of births ranged from 356 to 2790, with ten hospitals in this sample (69%) reporting more than 500 births.

**Table 1:** Factors related to postnatal accommodation and length of stay.

Factor	Private hospitals (n = 14)		Public hospitals*(n = 66)	
	**n**	**%**	**n**	**%**

Designated postnatal unit	13	93	29	44

	**median %**	**range**	**median %**	**range**

Postnatal bed occupancy	78	(41-97)	76	(0-99)
Length of stay ≥ four days	91	(79-100)	42	(0-82)**
Women aged ≥30 years	74	(57-88)	45	(25-63)
Australian-born women	84	(60-98)	93	(47-100)

* Data from original PinC review provided for comparison

** Public hospitals that had 60% or more women with ≥ four day stay were 8 rural hospitals and 2 very small metropolitan hospitals

### The hospital environment

Thirteen of the hospitals (all metropolitan and three regional hospitals) reported having a designated postnatal unit (Table 1). The hospital that did not have a designated unit reported that women receiving postnatal care were not accommodated in rooms with non-maternity patients. All provided single room accommodation, six had only single rooms, and no participating hospital had less than 50% single rooms. All the hospitals reported that accommodation was available for partners to stay overnight, with an average of 66% of the beds being suitable for a partner to stay (range 2% to 100%). Key informants reported that women commonly requested and preferred single rooms, with an expectation that private health insurance entitled them to a private room.

*A lot of our mothers...[are in their] mid to late thirties...and I think that has an influence on what they expect in terms of health care, especially because they have got private health insurance...they have an idea that this entitles them to a certain level of care and it goes along with the idea they should move straight into a double-bed room and that's often the first question they ask when they come through the door. I won't be sharing a room will I? (KI, Midwifery Manager, Metropolitan, 2003-1)*.

On average the bed occupancy was 78%, however it was higher in metropolitan hospitals (median 81%, range 41 to 97%) than regional hospitals (median 61%, range 50 to 71%) (Table 1). Most women (84%) stayed four days or more following the birth of their baby, and women in metropolitan hospitals were more likely to stay four days or more compared to women in regional hospitals (86% compared to 68%). While a small number of key informants expressed a desire for more flexibility in the length of stay, most believed that the longer length of stay available to women in private hospitals was ideal as it allowed women to rest and recover.

*Being private they're offered a four or five day stay and they have got a chance to recover from the birth, be fed, have their bed changed and not have to worry about anything like that for a few days. So it's a chance to recover from the birth (KI, Clinical midwife, Metropolitan, 2016-2)*.

Women's expectations regarding the hospital facilities and the physical environment of the postnatal unit was perceived by some care providers as an important consideration for women who choose private maternity care.

*Their [the parents] expectations are - they want a lovely room with a beautiful view and beautiful bed and they want a double bed and lovely meals and to be waited on hand and foot (KI, clinical midwife, metropolitan, 2003-2)*.

*When [hospital X] did their 'hotel' upgrade it killed the bookings at [our hospital]. We were forced to renovate. But the renovations were of physical facilities; there wasn't any change at all to the standard of nursery care, midwifery care, health care, or midwifery staff or obstetrics staff (KI, Obstetrician, Metropolitan, 2003-3)*.

#### Rooming-in and use of a well-baby nursery

The majority of hospitals (79%, 11/14) had a special care nursery and six (43%) also reported a well-baby nursery, both of which were staffed separately to the postnatal unit. Use of the well-baby nursery varied significantly between hospitals, that is, the estimated proportion of women who chose to use the option where their baby could be cared for while they rested ranged from 2% to 90%. Only four of the hospitals (29%) had a policy that babies should room-in with their mothers, although eight hospitals reported that in practice babies do stay with their mother all the time including overnight. All but one of the survey respondents commented on the issue of rooming in. Four hospital respondents considered requests by women for their baby to be cared for in the nursery as reasonable, implying that women (as private patients) had a right to make these requests. One respondent considered that rooming-in was not always appropriate:

*[I] don't think [rooming-in] should be enforced all of the time if a mother is really tired and needs a good rest. Have to weigh up pros and cons of fatigue and adequate milk supply/confidence (Survey, Regional, 2017)*.

Key informants reported that despite encouraging rooming-in, women often had strong expectations that their baby be taken to the nursery overnight.

*We try to encourage women to keep their babies with them overnight but we don't have a very high success rate. Women actually seem to prefer nursery care over night. Coming from women is a strong desire to have [their baby in the] nursery overnight so that they can get some sleep (KI, Clinical midwife, Metropolitan, 2016-2)*.

*Because the hospital has advertised it, there is an expectation in the community that women can have their baby in the nursery overnight (KI, Clinical midwife, Regional, 2009-2)*.

Thirteen hospitals (93%) also reported an option for women to leave their baby in the nursery if they chose to go out for a meal the night before discharge from hospital.

### Postnatal care practices

The provision of postnatal care practices regarding observation and documentation was relatively consistent in private hospitals across Victoria. All reported using care maps to guide postnatal care and most (85%) reported that maternal observations are routinely undertaken twice daily during the entire postnatal stay. Over half the hospitals (57%) also reported routinely recording neonatal observations twice daily for the entire postnatal stay.

#### Breastfeeding

Most hospitals (93%) reported having a breastfeeding policy; 71% provided post-discharge breastfeeding day-stay services or clinics; and 64% of hospitals had an option for individual visits from a lactation consultant available for women during the postnatal stay (Table 2). Only two hospitals reported that they were accredited as 'baby friendly' - the Baby Friendly Hospital Initiative (BFHI). The median proportion of women exclusively breastfeeding on discharge was reported to be 93% (range 82 to 98%).

**Table 2:** Breastfeeding outcomes and supports.

Factor	Private hospitals (n = 14)		Public hospitals* (n = 66)	
	**n**	**%**	**n**	**%**

Accredited as 'baby friendly' (BFHI])**	2	14	19	28
Hospital has breastfeeding policy	13	93	59	89
Optional individual LC*** consultation in hospital	9	64	33	50
Breastfeeding/day stay clinic	10	71	35	53

	**Median %**	**range**	**Median %**	**range**

Proportion women breastfeeding on discharge (private n = 10, public n = 49)	93	(82-98)	90	(51-100)

* Data from original PinC review provided here for comparison

** Baby Friendly Hospital Initiative

*** LC - Lactation consultant

#### Antenatal planning for the postnatal period

All except one hospital reported that women have an opportunity during pregnancy to discuss issues related to the postnatal period, either at the booking visit or during antenatal childbirth and parenting classes. Midwives were identified as the most likely care providers to discuss postnatal issues with women.

#### The role of the obstetrician in postnatal care

Obstetricians were reported by key informants to play a relatively minor role in postnatal care provision, usually a brief daily visit with women, and to be available if needed for any medical care.

*They [obstetricians] have a slightly peripheral role in postnatal care although they are there if we need them. Obviously they are available to their patients if they're needed, just advice really, and doing what we ask them to do. They're more than happy to leave it up to us and even if their patients ask them about breastfeeding they'll usually defer to us (KI, Midwife Manager, Metropolitan, 2019-1)*.

*Postnatally the midwives take charge, we [obstetricians] call in each day basically making sure everything is okay...it's very important that the obstetrician is not there all the time because he doesn't need to be when you have a midwife there (KI, Obstetrician, Metropolitan, 2003-3)*.

#### Visitors and rest

Eleven hospitals (79%) reported having a daily designated rest period. All had specific visiting times, although all hospitals also allowed fathers to visit at any time. Eight considered that visiting hours were adhered to most of the time, but comments suggested this was not always easy, e.g. 'We try!', 'Really try hard' and 'Staff find it difficult to enforce'. Only one respondent said that visiting hours were strictly adhered to.

#### Debriefing

Eight (57%) of the fourteen private hospitals routinely offer debriefing after caesarean birth. Three of these said that debriefing was on their care plan for day three after the birth. In most cases this means women are offered an opportunity to talk about their birth and to ask questions, rather than any formal debriefing being undertaken. However, two providers reported having a more formal debriefing opportunity for women if specifically requested.

### Staffing

The majority of midwives providing postnatal care in private hospitals worked part-time. In four hospitals, postnatal units were staffed only with core (permanent) postnatal staff, in five all midwives rotate through the postnatal units and other maternity areas and in three hospitals midwives also rotate through non-maternity areas. While hospitals reported that maternity care was organised in an attempt to provide continuity, there was an acknowledgement that at times this was difficult to achieve.

*[The] majority of staff [work] part time so [it is] difficult to provide continuity of care giver at all times. Attempts are made after handover so that staff familiar with particular patients, i.e. cared for them on previous day or provided care in birth suite, have those patients in their allocation for the day. Most of the time this system works quite well as all staff work in all areas (Survey, Regional, 2009)*.

The most common systems reported by private hospitals to guide daily staffing ratios were patient dependency systems (43%) or the hospital's own system (36%), which generally based staffing on a combination of numbers of women as well as acuity. Respondents reported adhering to their designated ratios 50% of the time. All private hospitals except one reported having a casual bank on which they are heavily dependent; the median number of bank shifts used in the postnatal unit in the month prior to the survey was 10 (range 1 to 21) (Table 3).

**Table 3:** Staffing of postnatal units.

Factor	Private hospitals (n = 14)		Public hospitals* (n = 66)	
	**median**	**range**	**median**	**range**

Midwives (EFT) providing postnatal care	31(15EFT)	(11-85)	15(10EFT)	(5-165)
Shifts per week covered by casual bank staff	10	(1-21)	3	(0.5-25)
Shifts per week covered by agency staff	2	(1-15)	1.75	(1-17)

	**n**	**%**	**n**	**%**

Hospitals employing midwives only	3	21	30	46
Hospitals employing Mothercraft nurses	6	43	10	15
Hospitals employing Division 2 nurses	5	36	18	27

* Data from original PinC review provided here for comparison

*We would have a bank nurse on each shift every day (Survey, Metropolitan, 2015)*.

*We use bank particularly in the afternoon shifts that are harder to fill. We have at least one sometimes two or three bank staff in the afternoon (KI, Clinical midwife, Metropolitan, 2016-2)*.

Eleven private hospitals also reported using agency staff to provide postnatal care and the median number of shifts in postnatal units covered by agency staff per week was two (range 1 to 15) (Table 3). Despite this dependency on casual bank and agency staff, most survey respondents (78%) felt that the postnatal units were adequately staffed. They reported few problems in retaining postnatal staff, although some hospitals reported having problems recruiting midwives, especially to cover night duty.

*We have been very lucky to recruit several staff on nurse bank lately. Staff tend to stay so retention is not a problem. The main issue is finding people to work night duty and weekends. There is a real shift to a 9-5 Mon-Fri mentality, family friendly hours, especially among new grads [sic], however hospitals do not work that way (Survey, Metropolitan, 2015)*.

Three of the hospitals reported that only midwives provided postnatal care (Table 3), with six reporting that mothercraft nurses and five reporting Division 2 nurses (known as enrolled nurses in other states) were also employed on their postnatal unit. Three reported having midwives, Division 2 nurses and mothercraft nurses employed to provide postnatal care. Concern was expressed by some key informants that care providers other than midwives were providing postnatal care:

*I think that with the high rates of caesareans you might get away with an RN looking after the Caesar patient and having a lactation consultant looking after the breastfeeding side. You might get away with it but there are some subtleties in postnatal care that I think only midwives would be able to pick up (KI, Obstetrician, Metropolitan, 2016-3)*.

### Aims of and barriers to achieving quality postnatal care

The aims of postnatal care were identified by key informants as the establishment and management of breastfeeding; the education of parents with the view of discharging a confident, competent mother; and the provision of rest and recovery for women following birth. However a number of issues were reported as barriers to achieving these aims including: the increased acuity of postnatal care; the prioritising of other areas over postnatal care; high midwife-to-women ratios; and the number and frequency of visitors. Three survey respondents specifically mentioned that it was often the women themselves who tell their visitors to come in at any time regardless of rest periods, and another said it was "an ongoing battle". It was felt that visitors reduced women's ability to adequately rest and also disrupted midwives' ability to fulfil their educative role during the postnatal stay.

*Visitors, well yes there is nothing we can do about that. They have visitors, they want visitors and they get exhausted and then they don't have the energy to feed the baby overnight and run into breastfeeding problems (KI, Midwifery Manager, Metropolitan, 2016-2)*.

*We try and get visitors to leave at the end of visiting times so that we can continue our care it really gets in the way of postnatal care (KI, Midwifery Manager, Metropolitan, 2003-1)*.

### Care options after hospital discharge

In Victoria, domiciliary postnatal care provided by a midwife is funded by the Department of Health for women who have public maternity care. Domiciliary care is not routinely provided by Victorian private hospitals, however one participating hospital reported that they did offer home visits to all women following discharge. A further three reported that domiciliary care is offered to women with identified needs (two of which noted that this included all primiparous women) and one hospital reported offering all primiparous women within a 20 km range a visit. Opinions on the provision of domiciliary care for women in private care varied; some hospitals felt it should be available to all women, whereas others considered that the longer length of stay available to women in private hospitals made the need for domiciliary care redundant.

*[The] private sector offers a wonderful, relaxing/flexible level of care, however [we are] prejudiced against by not being able to offer government assisted DOM care (Survey, Regional, 2017)*.

*Currently our women stay five days so domiciliary service is usually unnecessary (Survey, Metropolitan, 2006)*.

A number of private hospitals reported the introduction of early discharge options. Half the hospitals offer women early discharge 'packages' which include midwife home visits in most instances as well as some other 'incentives'. Two hospitals reported that they offer hotel postnatal care. The services offered in the hotel care 'package' include: a family stay at a four or five star hotel; daily visits from the obstetrician; the availability of lactation consultant and paediatrician visits; and access to a midwife 24 hours a day (with a ratio of one midwife to six women). All meals and hotel services are included. Although neither respondent provided an estimate of the proportion of women who take up the hotel care option, key informants reported that they believe the hotel postnatal care option is well received by the women who elect to use it.

*Women can go [to the hotel] day three or day four depending if they have had a normal birth or a Caesar. They go over there for a couple of nights and the midwives are working there with a ratio of 1 to 6 at the most. I think women are very happy with it as a service and it's rated very highly by women who have been there. I think it's a calmer, quieter environment for them. The rooms are quiet, they don't have people barging in and out all the time, [like] cleaners and us and kitchen [staff] and everything. They order their meals when they want to or don't want to and the midwife is just a phone call away. She's right there on the floor with them the whole time (KI, Midwifery Manager, Metropolitan, 2016-2)*.

Of the hospitals that offer a non-hotel care early discharge 'package', three reported that all women are offered this option, two that it was based on individual request, and one reported that women can opt for early discharge after their baby is born. Five respondents estimated the proportion of women that take-up the early discharge option; the median was 2% (range 1% to 14%). On average women taking up this early discharge option receive two midwife visits, although this was reported as being dependent on the time the woman spent in hospital. For example, in one hospital, women discharged 48 hours after an uncomplicated vaginal birth or 72 hours after a caesarean birth receive two home visits, of up to two hours per visit, from a hospital midwife known to the women. Another hospital offered midwife visits with early discharge until day five and two hospitals reported undefined postnatal 'packages'.

*We have a system of maternity care in the home where if women choose to go home on day three they will get two midwife home visits. It's geared at multis [sic] and it is reasonably popular (KI, Clinical midwife, Metropolitan, 2003-2)*.

Other postnatal supports that were reported and were available to all women at these hospitals included: coffee mornings for new mothers; 24-hour telephone service; the option of return to hospital for postnatal visits; breastfeeding clinics; house cleaning; meal vouchers at restaurants; free nappies; and physiotherapy consultations. Respondents reported that none of the early discharge initiatives had been formally evaluated.

### Women's satisfaction with postnatal care

Key informants identified aspects which they believed may have contributed to women's satisfaction with their postnatal care including: developing relationships with care providers especially in relation to: information, education and support with breastfeeding; communication, with women feeling that they are listened to; and the type of hospital environment provided in private hospitals, such as having the option for partners to stay overnight.

*I think the attitude of the staff, the empathy of the staff, the continuity of advice, the facilities, the standard of the meals and the response when they require help [makes a big difference to whether women are satisfied]. I think understanding when the women are really tired, that sometimes they just need a break and just to sit and talk (KI, Clinical midwife, Metropolitan, 2019-1)*.

*Listened to and looked after (KI, Obstetrician, Metropolitan, 2003-3)*.

## Discussion

This was the first review of postnatal care in Australian private hospitals and one of the few studies to explore the organisation and provision of postnatal care from the perspective of care providers. The review identified a number of key organisational differences in the provision of postnatal care between private and public hospitals. Compared to public hospitals, private hospitals are more likely to provide postnatal care in separate units configured with single rooms; accommodate partners overnight; offer well-baby nurseries; and have a longer length of postnatal stay. Private hospitals are more likely to use a casual workforce and staff postnatal units with non-midwives. Only 29% of responding hospitals had a policy of rooming-in and only two were accredited as BFHI. Care provision following discharge varies, with no consistent domiciliary service provision in the private sector.

Given the existing evidence that women in the private sector are consistently more satisfied with hospital postnatal care,[3] survey respondents and key informants identified aspects they considered may contribute to this, including: a longer length of stay; the aesthetic environment of the postnatal unit (single rooms with more privacy); having partners stay overnight; and the option of babies being cared for in well-baby nurseries. These views of what aspects contribute to women's satisfaction are resonant with recent research conducted in Sweden, which showed women's negative experiences of postpartum care were related to perceptions of insufficient staffing; an inflexible length of stay, especially for first time mothers; a poor postnatal physical environment; the inability of partners to stay overnight; and problems associated with the provision of postpartum care in a hotel[4].

**Figure 1:**
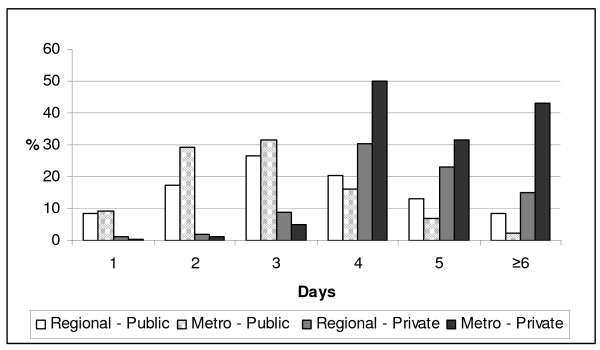
**Length of stay in public and private hospitals**.

Length of stay following birth has dramatically declined over the past two decades in Victorian public hospitals, a trend reflected internationally[31]. Most women receiving postnatal care in private hospitals stayed four days or more after the birth of their baby compared to public hospitals where, despite some variation related to geographic location, only 20% of women stayed for four days or more (Figure 1)[32]. The transition to parenting can be filled with anxiety for some women and their partners, and recent evidence suggests the reassurance gained from a longer length of stay - having the close proximity of midwives in the early postnatal period, especially for first time mothers is important to some women[4,26,33,34].

While the key informants reported that they believed women were satisfied with the early discharge initiatives such as hotel care and midwife home visits, only a small number of women took up these options. There is little evidence regarding Australian women's experiences and outcomes of these models of postnatal care, however the provision of postnatal care at home has been offered in the Netherlands for well over a century[31]. Hotel postnatal care has been tried in the United States as an economic alternative to hospital care [35] and in Sweden in an effort to provide a more family like environment[36]. We identified limited evaluation of hotel postnatal hotel care, but women in Sweden, particularly those having their first baby, reported hotel-based postpartum care to be 'impersonal, isolated and inappropriate for the new born baby'[[4]:p.7].

The aims of postnatal care as described by care providers in private hospitals (the establishment and management of breastfeeding, parental education, and the provision of rest and recovery for women following birth) were the same as those reported in a review of public postnatal care providers in Victoria[22]. Similarly many of the problems reported by midwifery managers providing postnatal care in private hospitals were the same as reported in the public review[19-22].

Key informants reported the establishment and management of breastfeeding as an aim of postnatal care and the survey showed high rates of breastfeeding among women at discharge. However, only 29% of responding private hospitals had a policy of rooming-in and many babies were routinely separated from their mothers, especially overnight in a well-baby nursery. The rationale for maintaining well-baby nurseries is that they enable women to rest and recover from the birth. On the other hand, almost all public hospitals (99%) reported that it was standard practice for infants to be with their mothers at all times compared to less than a third of private hospitals, as only one public hospital still maintains a well-baby nursery.

Infant rooming-in is one of the ten steps in BFHI which is an evidence-based initiative that improves the successful establishment of breastfeeding[37]. In this study, only 2% of private hospitals had BFHI accreditation. In Australia, despite high breastfeeding initiation rates, the proportion of women feeding any breast milk at six months is less than 50%, although women who choose private care are more likely to initiate and maintain breastfeeding[38]. It may be that increased compliance with the BFHI recommendations could increase breastfeeding rates further in this group of women.

The provision of private hospital postnatal care, like public hospital postnatal care, is characterised by high bed occupancy and a lack of midwifery staff to provide care in a very busy environment. While private and public hospitals both report difficulties in attracting midwives to staff postnatal care, private hospitals rely heavily on part-time, non-core, and non-midwifery staff, and have a greater dependency on casual bank and agency midwives. Despite the increased use of private hospitals since the introduction of Australian national government legislation to take up private health insurance[39] and more 'patient generated' revenue reported by private hospitals,[11,40] there has been a decrease in the total number of nursing and midwifery staff employed in the private hospital sector[12].

The shortage of midwives is a recognised problem in Australia and expected to continue[41]. Recruiting and retaining midwives is an ongoing problem reportedly related to midwives' perceptions of a lack of professional recognition, stress and workload issues, as well as limited opportunities for midwives to practice as primary carers and provide continuity of care to women[42,43]. These issues were identified in the review of public hospital postnatal care[20,22] and have also been cited in international literature[24,44,45]. Recent research from Australia suggests midwives working in postnatal care may feel a sense of 'grief' associated with changes to the organisation of postnatal care, as their practice has been further constrained by 'institutional policy and medicalised routines'.[46:p5] Midwives are particularly concerned at the shortening length of postnatal stay in public hospitals[46] and this concern is echoed in recent research into women's views on different postnatal care packages[33,34].

Care providers in this study reported that they believed women choosing private care have expectations regarding the type of postnatal accommodation and that women approach maternity care as a consumer with a 'right to choose'. Choice has increasingly been recognised as a consumer concern in health care in Australia and having private health insurance allows individuals the choice of medical specialist and hospital[47]. This view is supported by research into private health insurance membership in Australia[48,49] and elsewhere[50,51] which has been shown to be associated with ideas of entitlement and perceptions about the service environment and delivery, as well as the quality of care. The aesthetic quality of postnatal care has been reported as a determinant in women's satisfaction with postnatal care[1]. The impact and relevance of choice may well influence women's expectations and overall satisfaction with maternity care, irrespective of higher rates of obstetric interventions found in private maternity care[16-18].

While information on mode of birth was not sought in the postal survey nor specifically raised by key informants, it is likely to have an impact on several of the findings identified in this paper. Some of the issues raised in relation to the provision of postnatal care included the busyness of postnatal units, the increased workload for staff, the longer length of stay and the high bed occupancy, all of which may be impacted on by the high caesarean birth rate among women receiving maternity care in the private sector[52].

Limitations of this study include the 74% response rate to the survey by private hospitals. It is unknown what the views of care providers in those hospitals are and generalisations about all private hospital postnatal care cannot be made. However, the responses from hospitals that did respond (a range of large and small hospitals) coupled with the majority (96%) of hospitals participating in the public review, provides a relatively complete picture of postnatal care in Victoria from the perspective of care providers. As detailed in the paper, this study did not examine women's views of postnatal care however these views have been explored elsewhere and this was not the aim of this study.

## Conclusions

This review of private postnatal care in Victoria, Australia demonstrated both similarities and differences in the organisation and provision of postnatal care compared to postnatal care in public hospitals. Key differences related to the organisational and aesthetic aspects of service provision aspects rather than the delivery of postnatal care. Compared to public hospitals, private hospitals more often provide single room accommodation and well-baby nurseries, can accommodate partner's over-night, and enable women to have a longer length of stay in hospital following birth. However, private hospitals have the same problems adequately staffing postnatal units as public hospitals and are more dependent on non-midwifery staff, casual and bank midwives to provide postnatal care than public hospitals. Only 29% of private hospitals have a policy on rooming-in and only 2% are accredited as baby-friendly.

Key messages emerging from both reviews include the need to review staffing levels and develop alternative approaches to postnatal care to improve women's satisfaction with this episode of care. The results may be timely in light of major changes in the provision of this episode of care in Victorian public hospitals, where the length of the postnatal hospital stay is being further reduced. However, the findings support the need for further research. In particular we recommend studies that provide evidence for practice in the postnatal setting, as well as more indepth explorations of women's views and experiences of postnatal care, and the relationship of these with maternal and infant health outcomes.

## Competing interests

The authors declare that they have no competing interest.

## Authors' contributions

The study was conceived and designed by HMcL, DF and JY; DF and HMcL undertook the survey data analysis; JR, LP and HMcL conducted the key informant interviews and JR and LP analysed the data. All authors contributed to the manuscript and read and approved the final version

## Pre-publication history

The pre-publication history for this paper can be accessed here:

http://www.biomedcentral.com/1471-2393/10/26/prepub

## Supplementary Material

Additional file 1 **Draft Survey**. A draft copy of the PinC Private survey. Click here for file

Additional file 2 **Glossary of Terms**. A list of descriptive terms used in the paper which may be specific to Australia. Click here for file
