# Supplementary material for: A statewide review of postnatal care in private hospitals in Victoria, Australia
Source: BMC Pregnancy Childbirth. 2010 May 28;10:26. doi: 10.1186/1471-2393-10-26 (PMC2891607; doi:10.1186/1471-2393-10-26)
Supplement: Additional file 1 — Draft Survey. A draft copy of the PinC Private survey. [file 1471-2393-10-26-S1.PDF]

CONFIDENTIAL

Hospital ID: ☐☐☐☐

PinC Private
A Review

A review of postnatal care in Victorian private hospitals

Thank you for taking the time to participate in the PinC Private Review. The attached questionnaire requires you to tick appropriate response boxes or to write comments in the spaces provided. We have also asked that you refer, where applicable, to your Perinatal Data Collection Unit (PDCU) hospital profile 2004.

If you have any questions about the study or the questionnaire please call (03) 8341 8500 and ask to speak to one of the PinC Private team members.

Please note that questions appear on both sides of the pages in this questionnaire. When completed please return the survey and the requested documentation in the stamped addressed envelope to:

PinC Private: a review of postnatal care in Victorian private hospitals
Mother & Child Health Research
La Trobe University
251 Faraday Street
CARLTON, Vic 3053

For office use only

Date sent ______/______/______

Date received ______/______/______

DO NOT COPY

A Your maternity service

A1 (a)Where is your hospital located? *(please tick only one)*

1☐ Rural

2☐ Regional

3☐ Metropolitan

4☐ Other *(please describe)*

____

____

(b)Regarding neonatal services, is your maternity unit(s)?

1☐ Level 1

2☐ Level 2

3☐ Not applicable *(no birth suite) (Go to QA3a)*

A2 Referring to your Perinatal Data Collection Unit (PDCU) 'Hospital Profile <u>2004</u>', how many women gave birth in your hospital in 2004?

_____________ **Women**

A3 Referring to your 'Hospital Profile 2004' please indicate how many women were in the following age categories:

Age category	***Number of women***	***% of all women giving birth in 2004 at your hospital***
<20 yrs		
20-24 yrs		
25-29 yrs		
30-34 yrs		

A3a Could you estimate the number of women who booked for postnatal care <u>only</u> at your hospital in 2004? (ie had their antenatal and intrapartum elsewhere?)

1☐ None

2☐ _________**Women**

DO NOT COPY

A3b Can you estimate the number of babies that were transferred to your care from a Level 3 (tertiary) hospital in 2004?

1☐ None (Go to question A4)

2☐ __________**Babies**

A3c When a baby is transferred from a tertiary hospital, is the baby's mother also admitted?

1☐ Yes

2☐ No

3☐ Other (please describe)

____

____

A4 Referring to your 'Hospital Profile 2004' please indicate the five main country of birth categories of the women attending your hospital in that year.

Country of birth	*Number of women*	*% of all women giving birth in 2004 at your hospital*
1. Australia		
2.		
3.		
4.		
5.		

DO NOT COPY

A5 Referring to your PDCU 'Hospital Profile 2004' please indicate the average length of stay for women who gave birth in your hospital in 2004.

Postnatal length of stay	*Number*	*Percent*
1 day		
2 days		
3 days		
4 days		
5 days		
6 days or more		

A6 Can you <u>estimate</u> the overall average bed occupancy for <u>postnatal care</u> in your unit(s) for <u>May 2006?</u>

1☐ _____________ %

2☐ unable to estimate

A7 (a) Do you have a designated postnatal unit(s)? *(not usually shared with non-maternity patients)*

1☐ Yes *(please go to A7c)*

2☐ No

3☐ Other *(please describe)*

(b) If 'No', are women who have given birth accommodated in the same rooms with non-maternity patients?

1☐ Yes *(please describe patient mix)*

2☐ No

3☐ Other *(please describe)*

(c)If you have a designated postnatal unit(s), how is the unit(s) configured? *(please tick all that apply)*

1☐ Single room(s)

2☐ 2 bed room(s)

3☐ 3 bed room(s)

4☐ 4 or more bed rooms(s)

5☐ Other *(please describe (eg single room(s) with double bed))*

(d) Can you estimate what proportion of your overall postnatal unit comprises single rooms?

1☐ ……..%

2☐ none

(e) Do you have provision for the woman's partner (or other person) to stay in hospital postnatal unit overnight?

1☐ Yes

2☐ No

(f) If yes, in what proportion of rooms is it possible for the partner/other to stay?

DO NOT COPY

Number with partner beds _______

Total number of beds in unit ________

B Models of care

B1 What models of *antenatal* care are available to women booking for birth at your hospital?
(please tick all that apply)

1☐ Postnatal care only (*please go to Section C*)

2☐ Care with a private obstetrician

3☐ Care with a private GP/Obstetrician (antenatal, intrapartum, postnatal care)

4☐ Combined care *(GP/Obstetrician antenatal care not provided at the hospital, but booking for public intrapartum and postnatal care)*

5☐ Shared care *(including some check-ups at the hospital and some with a GP or obstetrician in their private rooms)*

6☐ Birth Centre *(discrete unit offering antenatal, intrapartum and postnatal care)*

7☐ Public obstetric care

8☐ Other *(please describe)*

____

____

____

B2 Is there an option for women attending your hospital to have some antenatal care with a midwife?

1☐ Yes

2☐ No

Please comment

____

____

____

DO NOT COPY

C Staffing

C1 This section, (C1) relates to midwives permanently employed by the hospital, not casual or bank staff or student midwives. 'Rotating' refers to those midwives that rotate either within the maternity unit(s) or between the postnatal area and other non-maternity areas of the hospital. Questions regarding bank and casual staff and student midwives are asked about later in this section.

(a) In total how many midwives, are currently employed by your hospital to provide in-hospital postnatal care? *(include core and rotating staff)*

Total **number** of midwives ____________ ☐☐☐

(b) What EFT of midwives is currently employed to provide in-hospital postnatal care?

Total **EFT** of midwives ____________ ☐☐☐

(c) Of those postnatal midwives can you estimate the number that work in each of the following capacities?

1☐ Core staff part-time *(<38 hrs per week)* ____________ ☐☐☐

2☐ Core staff full-time *(38 hrs per week)* ____________ ☐☐☐

3☐ Rotating staff full-time *(postnatal & other maternity areas)* ____________ ☐☐☐

4☐ Rotating staff part-time *(postnatal & other maternity areas)* ____________ ☐☐☐

5☐ Rotating staff full-time *(postnatal & non-maternity areas)* ____________ ☐☐☐

6☐ Rotating staff part-time *(postnatal & non-maternity areas)* ____________ ☐☐☐

C2 On average how many midwives work in postnatal care each shift? *(Including all categories of midwives, eg permanent, bank, casual. Excluding student midwives)*

1☐ AM ____________ ☐☐

2☐ PM ____________ ☐☐

3☐ ND ____________ ☐☐

4☐ Other *(please describe)* ☐☐

____ ☐☐

____ ☐☐

DO NOT COPY

C3 (a) Do you have student midwives undertaking placement in your postnatal unit(s)?

1☐ Yes

2☐ No *(please go to C5)*

☐☐

(b) Can you detail the type of midwifery students involved in postnatal care at your hospital *(please tick all that apply)*

Student	***Paid***	***Unpaid***
**Undergraduate**<br>Bachelor Midwifery/ Bachelor Nursing & Midwifery		
**Postgraduate**<br>RN undertaking Postgraduate diploma in midwifery		

☐☐

(c) When on placement are student midwives supernumerary?

1☐ Yes

2☐ No

3☐ Other *(please describe)*

____

____

☐☐

(d) If you have paid student midwives in your postnatal unit(s), what is the average ratio of midwives to students?

Number of midwives ________ to **number** of students________

☐☐/☐☐

(e) What percentage of patient load do the student midwives take compared to registered midwives?

1☐ Full patient load

2☐ 75% of patient load

3☐ Other *(please describe)*

____

____

☐☐

DO NOT COPY

C4 What other nursing staff work <u>regularly</u> on the postnatal unit(s)? *(tick all that apply)*

1☐ Mothercraft nurses

2☐ Enrolled nurses *(Division 2)*

3☐ Registered nurses *(Division 1)* not endorsed as midwives

4☐ None

5☐ Other *(please describe)*

____

____

C5 (a) How do you determine staff/patient ratios in your postnatal unit(s)?

1☐ ANF ratios

2☐ Patient dependency system

3☐ Own hospital ratios (different from ANF ratios)

4☐ Other *(please describe)*

____

____

(b) If you work on ratios, what is the ratio of nurse/midwife to woman on the postnatal ward?

<u>AM shift</u> **No.** of nurses/midwives: ________ to **<u>no.</u>** of women:________

<u>PM Shift</u> **No.** of nurses/midwives: ________ to **<u>no.</u>** of women:________

<u>Night shift</u> **No.** of nurses/midwives: ________ to **<u>no.</u>** of women:________

(c) Can you <u>estimate</u> the number of shifts per week where staff-patient ratios were NOT adhered to in May 2006?

<u>Number</u> of shifts/week _____________

C6 (a) Does your hospital have a nurse/midwife casual bank/pool?

1☐ Yes

2☐ No *(please go to C7)*

(b) Could you approximate how many shifts per week the <u>postnatal unit(s) in your hospital</u> had at least one <u>nurse bank/pool staff</u> working (including night shifts) in May 2006?

<u>Number</u> of shifts per week _____________

DO NOT COPY

C7 **(a) Does your hospital use the services of a nursing agency?**

1☐ Yes

2☐ No *(please go to C8)*

(b) Could you approximate how many shifts per week the postnatal unit(s) in your hospital had at least one agency staff working in May 2006?

Number of shifts per week ____________

(c) When overall staffing levels are low, or you are very busy, is the birth suite prioritised over the postnatal unit(s) in terms of staffing? *(agency/bank staff are more likely to be used in postnatal unit)*

1☐ Yes

2☐ No

3☐ Not applicable *(no birth suite)*

4☐ Other *(please describe)*

____

C8 **In general do you think your postnatal area is adequately staffed?**
Please comment

____

____

____

C9 **Please comment on any issues you have with recruitment and retention of midwives at your hospital.**

____

____

____

____

____

DO NOT COPY

C10 What resources are available to women during their postnatal stay? *(please tick all that apply)*

Resources	***Yes***	***No***
Lactation consultant(s)		
Physiotherapist(s)		
In-house interpreter(s)		
Social worker(s)		
Dietician(s)		
Counsellor(s)		
Other *(please describe)*		

C11 Are there any further comments you wish to make with regard to staffing?

C12 (a) How is handover given in the postnatal unit(s) in your hospital? *(please tick all that apply)*

1☐ Verbal handover with all on-coming staff

2☐ Verbal handover with individual on-coming staff

3☐ Audiotaped handover

4☐ Other *(please describe)*

(b) Who gives the handover? *(please tick all that apply)*

1☐ Person in-charge on shift

2☐ Person caring for women on shift

3☐ Other (please describe)

DO NOT COPY

D Documentation

D1 What documentation does your unit use in relation to postnatal care? Could you please attach a copy of this documentation? *(please tick all that apply, and include other documentation if applicable)*

1☐ Care map/pathway

2☐ Progress notes

3☐ Medication chart *(maternal)*

4☐ Medication chart *(neonatal)*

5☐ Maternal observation chart

6☐ Neonatal observation chart

7☐ Documentation of infant feeding

8☐ Breast feeding chart

9☐ Postnatal protocols

10☐ Postnatal guidelines

11☐ Discharge planning document

12☐ Risk assessment tool *(please describe)*

13☐ Other *(please describe)*

DO NOT COPY

D2 (a) If care maps are used, do staff use them to guide/ plan/ provide care?

1☐ Yes

2☐ No

3☐ Not applicable

(b) Do you have any comments you wish to make in relation to the use of care maps in your hospital?

D3 Do you have any further comments to make with regard to documentation in the postnatal area?

DO NOT COPY

E Rooming in

E1 **(a) Do you have a hospital policy of rooming-in?** *(ie. that babies stay with their mothers all the time, overnight etc.)*

1☐ Yes

2☐ No

3☐ Other *(please describe)*

(b)In general, is it practice that babies stay with their mothers all the time, including overnight?

1☐ Yes

2☐ No

3☐ Other *(please describe)*

E2 **(a) Does your hospital have a nursery for babies requiring special care (as distinct from a separate nursery for well babies)?**

1☐ Yes

2☐ No (Go to E2c)

3☐ Other *(please describe)*

(b) Is your nursery (for babies requiring special care) staffed separately?

1☐ Yes

2☐ No

3☐ Other *(please describe)*

DO NOT COPY

(c) Does your hospital have a nursery for well babies? (ie babies not needing special care)

1☐ Yes

2☐ No (Go to E2f)

3☐ Other *(please describe)*

____

____

____

(d) Is your nursery (for well babies who do not require special care) staffed separately?

1☐ Yes

2☐ No

3☐ Other *(please describe)*

____

____

____

(e) If your nursery for well babies is staffed separately, who is it staffed by? (eg midwives, mothercraft nurses)

____

(f) Can women ask that their baby go to the nursery/other area to be looked after while they rest?

1☐ Yes

2☐ No (Go to E3)

3☐ Other *(please describe)*

____

____

(g) Is the well baby nursery separate to the special care nursery?

1☐ Yes

2☐ No (Go to E3)

3☐ Other *(please describe)*

____

____

(h) If women have the option of having their baby go to the nursery/other area to be looked after while they rest, can you estimate what proportion of women take this up during their postnatal stay?

_________%

DO NOT COPY

E3 **Are there any comments you wish to make about rooming-in?**

E4 **(a)Is there an option for women to leave their baby in hospital care whilst they go out for a meal/outing prior to being discharged home?**

1☐ Yes

2☐ No. (Go to F1)

3☐ Other *(please describe)*

(b)If yes, can you estimate what proportion of women take up this option?

_______%

F Visitors and rest

F1 **(a)What are the visiting hours in your unit(s)?** *(please specify the hours visitors can come)*

(b) Are visiting hours strictly adhered to?

1☐ Yes, *(please comment)*

2☐ No

DO NOT COPY

F2 **(a) Can partners visit at any time?**

1☐ Yes

2☐ No

3☐ Other *(please describe)*

(b) Can a partner (or person of choice) stay with the woman overnight?

1☐ Yes

2☐ No

3☐ Other *(please describe)*

F3 **(a) Is there a designated 'rest' period?**

1☐ Yes

2☐ No *(please go to F4)*

3☐ Other *(please describe)*

(b) Is the rest period generally adhered to?

1☐ Yes

2☐ No

3☐ Other *(please describe)*

(b) What activities are restricted during the rest period?

F4 **Do you have any further comments in relation to visitors and rest?**

DO NOT COPY

G Postnatal observations

G1 What is your policy on frequency of <u>routine maternal observations</u>, not including the initial post birth observations, for women who have had a <u>normal vaginal birth</u>? *(if there are no risk factors indicating more frequent observations)*

Frequency	*Day 1*	*Day 2*	*Day 3*	*>Day 3*
Daily				
Twice daily				
Not routinely done *(please go to G3)*				

G2 If <u>maternal</u> observations are routinely undertaken, what is measured or noted? *(please tick all that apply)*

Observation	*Measured/ noted*	*Asked about*
1☐ Pulse		n/a
2☐ Temperature		n/a
3☐ Blood pressure		n/a
4☐ Fundal height/ involution		
5☐ Lochia		
6☐ Breasts		
7☐ Urinary function		
8☐ Bowels		
9☐ Perineum inspected *(if applicable)*		
10☐ Caesarean wound *(if applicable)*		
11☐ Legs *(DVT/oedema)*		
12☐ Emotional wellbeing/mood		
13☐ Interaction with baby *(attachment)*		
14☐ Other *(please describe)*		

DO NOT COPY

G3 How often are neonatal vital signs, eg. temperature, respirations & heart rate, routinely done on the postnatal ward? *(if there are no risk factors indicating more frequent observations)*

1☐ Daily

2☐ Twice daily

3☐ Not done routinely

4☐ Other *(please describe)*

G4 What other neonatal observations are routinely measured or noted? *(please tick all that apply)*

1☐ Bowels

2☐ Urine output

3☐ Feeding

4☐ Umbilical cord

5☐ Weight

6☐ Skin colour

7☐ Eyes

8☐ Mouth

9☐ Other *(please describe)*

DO NOT COPY

G5 **a) Is a pre-discharge neonatal check (including hips, heart sounds) routinely undertaken on babies prior to discharge?**

1☐ Yes

2☐ No (Go to G6a)

3☐ Other *(please describe)*

b) If yes, who <u>usually</u> undertakes the pre-discharge <u>neonatal</u> check in your hospital?

1☐ Midwife

2☐ Obstetrician

3☐ GP

4☐ Paediatrician

5☐ Other *(please describe)*

G6 **(a) Are women routinely <u>seen by</u> their doctor while in the postnatal ward prior to discharge?**

1☐ Yes

2☐ No

3☐ Other *(please describe)*

(b) Is it hospital policy that a doctor conducts a postnatal discharge check on women?

1☐ Yes

2☐ No

3☐ Other *(please describe)*

(c) Are babies on the postnatal ward routinely seen by a paediatrician?

1☐ Yes

2☐ No

3☐ Other *(please describe)*

DO NOT COPY

H Continuity

H1 We are interested in whether postnatal unit(s) are organised in a way that attempts to provide continuity of caregiver. Please describe if and how your unit(s)does this?

I Other issues in postnatal care

I 1 (a) From the 'Hospital Profile 2004' please indicate what proportion of women giving birth were of non-English speaking background that year? *(found later in PDCU report)*

1☐ ……………………………%

2☐ Not available

3☐ Not applicable (no births)

(b) What services are available to women of non-English speaking background during their postnatal stay? *(please tick all that apply)*

1☐ Hospital interpreter services

2☐ Telephone interpreter services

3☐ Written information in languages other than English

4☐ Other *(please describe)*

DO NOT COPY

I 2 **(a) From the 'Hospital profile 2004' please indicate what proportion of women giving birth were indigenous?**

1☐ Indigenous……………….%

2☐ Non-indigenous……………………..%

3☐ Not applicable (no births)

(b) Do you have any services that are available specifically for indigenous women in the postnatal unit(s)? *(please describe)*

I 3 **(a) Is your hospital an accredited "baby friendly" hospital?**

1☐ Yes *(please go to I 4)*

2☐ No

(b) Do you intend to apply for this accreditation?

1☐ Yes

2☐ No

3☐ Don't know

I 4 **(a) What do you estimate was the proportion of women breastfeeding at discharge in May 2006?**

<u>Exclusive</u> breastfeeding…………….%

<u>Any</u> breastfeeding………………….% *(includes those exclusively breastfeeding)*

(b) Are these breastfeeding data routinely collected?

1☐ Yes

2☐ No

3☐ Other (*please comment*)

DO NOT COPY

I 5 Does your postnatal unit(s) have any of the following? *(please tick all that apply)*

1☐ Breastfeeding policy

2☐ Ongoing education on breastfeeding (for staff)

3☐ Midwives who are qualified as lactation consultants

4☐ Optional individual lactation consultations whilst inpatient

5☐ Breastfeeding / day stay clinic

6☐ Other *(please describe)*

I 6 (a) At your hospital is 'debriefing' regarding labour and birth <u>routinely</u> offered to women in the postnatal unit(s) following birth?

1☐ Yes

2☐ No

3☐ Other *(please describe)*

(b) If 'Yes', what does this involve? *(Please describe)*

DO NOT COPY

I 7 Some hospitals have women attending the postnatal unit(s) who have additional needs (ie chemical dependency, baby in SCN/NICU, very young women). Does your hospital provide any specific programs for women with additional needs?

J Preparation for the postnatal period & discharge home

J1 (a) Does your hospital provide opportunities to discuss postnatal issues with women <u>during pregnancy?</u> *(eg breast feeding, postnatal depression, SIDS, social support)*

1☐ Yes

2☐ No *(please go to J2)*

3☐ Other *(please describe)*

(b) When does this discussion take place?

(c) Who do women have this discussion with? *(please tick all that apply)*

1☐ Midwife

2☐ GP

3☐ Obstetrician

4☐ Other *(please describe)*

(d) Does this include a discussion <u>prior to birth</u> about what to expect during their postnatal stay?

1☐ Yes

2☐ No

3☐ Other

DO NOT COPY

(a) If care maps/pathways are used, is a copy of the care map given to women at this time? (ie. prior to admission to hospital)

J2

1☐ Yes *(please go to J 3)*

2☐ No, given at another time *(please go to J2b)*

3☐ Care maps/pathways not given to women at any time *(please go to J3)*

4☐ Not applicable as care maps not used *(please go to J3)*

(b) When do they receive it?

1☐ Another time in the antenatal period

2☐ In the postnatal ward

3☐ Other *(please describe)*

J3 **(a) What educational sessions are women offered while in hospital during their postnatal stay?** *(please tick all that apply)*

Education type	*Individual discussion*	*Group session*
Community resources		
SIDS		
Settling techniques		
Parenting skills		
Pelvic floor exercises		
Physiotherapy		
Breast feeding		
Care of newborn		
Baby behaviour		
Other *(please describe)*		

J4 **(a) Are women provided with any written information about the postnatal period?**

DO NOT COPY

1☐ Yes

2☐ No *(please go to section Section J5)*

3☐ Other *(please describe)*

(b)Please detail <u>what information</u> is provided and <u>when</u>? *(during pregnancy, following the birth).*

(c) If possible, we would appreciate a copy of all booklets, brochures and pamphlets that are provided to women related to postnatal care. *(Please attach these to the completed survey).*

1☐ Consumer information attached

2☐ Unable to attach consumer information

DO NOT COPY

J5 **Are there any issues in relation to referral to community supports/services that you would like to comment on?**

1☐ No

2☐ Yes *(please describe)*

K Staff development

K1 **Could you please list the postnatal topics that were presented or discussed as part of in-service education in the last 12 months?**

DO NOT COPY

K2 Are there any areas of postnatal care that you think should be the topic of more in-service education? If so, what topics? *(please describe)*

L Care following discharge

L1 (a) How are decisions made regarding the provision of domiciliary care to each individual woman?

1☐ All women are offered a visit

2☐ Visits offered only to those women with identified need *(short length of stay, poorer maternal or infant outcomes, sick infant, infant feeding problems)*

3☐ Dom care is not routinely offered at this hospital *(please go to L4)*

4☐ Other *(please describe)*

(b) Of the women who receive domiciliary visits, how many do they receive?

	0 Visit	*1 Visit*	*2 Visits*	*3 Visits*	*More*
**Primiparae**					
**Multiparae**					

DO NOT COPY

L2 Following discharge home, after a normal length of stay, do women in your care receive domiciliary visits from: *(please tick all that apply)*

1☐ Your hospital midwife domiciliary service

2☐ Another hospitals domiciliary service

3☐ The Royal District Nursing Service

4☐ Private nursing agencies

5☐ Other *(please describe)*

6☐ N/A

L3 a) If your hospital provides domiciliary services please describe who provides this care? *(eg discrete domiciliary staff, by postnatal unit midwives, other)*

L4 (a) Does your hospital offer women the option of postnatal care in a hotel?

1☐ Yes

2☐ No (Go to L5)

3☐ Other *(please describe)*

DO NOT COPY

(b) How are decisions made regarding a woman's access to/use of hotel care?

1☐ All women are offered this facility

2☐ Other (please describe)

(b) Under what circumstances would a woman be excluded from hotel care?

(c) What support is offered to women who choose this option? (e.g. a midwife available 24 hours? Lactation consultant? Obstetrician visits?)

(d) Can you estimate what proportion of women take up the option of hotel care?

………..% Overall

………..% of those who are eligible

(e) What is the usual ratio of midwives to women in hotel care?

Number of midwives ________ to **number** of women ________

L5 **(a) Does your hospital offer women the option of early discharge with daily midwife visits to the woman's home?**

DO NOT COPY

1☐ Yes. Please describe:

2☐ No (Go to L6)

3☐ Other *(please describe)*

DO NOT COPY

(b) How are decisions made regarding early discharge home?

1☐ All women are offered this option

2☐ Other *(please describe)*

(c) **Can you estimate what proportion of women take up this option?**

………..% Overall

………..% of those who are eligible

(d) How many home midwife visits do women generally receive with this option?

1☐ 1 visit

2☐ 2 visits

3☐ 3 visits

4☐ >3 visits

5☐ Number of visits depends on the length of time women spend in hospital or on a number of other factors (maternal request, family situation) Please provide details:

6☐ Other *(please describe)*

DO NOT COPY

L6 (a) Does the hospital offer women any other discharge supports such as meals, child care, cleaning etc?

1☐ No

2☐ Yes (*please describe, and also comment on how this is funded)*

(b) Do you have any other comments on discharge processes or supports?

(c) Over the past two years, has your hospital changed/reduced any services offered to women following discharge?

1☐ No

2☐ Yes (Please describe)

DO NOT COPY

M To finish….

M1 Are there any areas of postnatal care that you regularly audit in your hospital? *(eg. breast feeding initiation rates)*

1☐ Yes *(please describe what and how often)*

2☐ No

M2 Is there anything else would you like to tell us about the provision of postnatal care? We would be interested in your views of the organisation of care, limitations, positive aspects and any new initiatives.

DO NOT COPY

Thank you very much for taking the time to fill in this survey. We appreciate your involvement in PinC and the time you have taken in completing this questionnaire.
As we may need to clarify some of your responses would you be happy to provide us with your name and contact telephone number? (this is optional)

Name:…………………………….

Phone number:……………………

Please read the accompanying letter regarding the key informant interviews and complete the checklist on the back page. Thank you once again.

DO NOT COPY

Would your organisation be willing to participate further in this review of postnatal care in the private sector? We are seeking to interview 15 caregivers across the state.

The second stage of this project will be to conduct interviews with care providers. This will generate a more in-depth understanding of care from the caregiver's perspective, especially around the complexities and limitations of providing postnatal care, any barriers that prevent caregivers from providing care in the way that they would like to; and any suggestions for innovations aimed at improving the provision of care.

We have randomly selected maternity units across Victoria to participate in this component of the review and your hospital is one of those selected. We would like to invite **two midwives** (*Maternity Unit Manager/Associate Unit Manager, and a clinical midwife (Grade 2 or CNS)* and **one medical practitioner** who provides maternity care in your hospital to an interview with a member of the research team at a time and place of their convenience. Interviews will take around 45 minutes.

Interviewees will be given the opportunity to discuss their views and experiences of postnatal care in the private hospital setting. The organisation and the individual interviewees will be provided with a summary of the findings from both the hospital survey and the interviews with key informants. This will only be a group summary from all hospitals combined, no individual data will be provided.

This interview process is entirely voluntary and the information provided by the interviewees is strictly confidential. Should your organisation not wish to participate please contact one of the research team.

If you would like any more information, please do not hesitate to ring one of the PinC Private team on (03) 8341 8500.

If you are willing to allow us to approach midwives and a doctor in your organisation, please fill out the accompanying form. Before inserting individual contact details, please ensure that the staff nominated will be happy for this information to be passed on to the research team.

DO NOT COPY

Hospital ID: ☐ ☐ ☐ ☐
(office use only)

**Potential key informant 1 (NUM/ANUM)**	**Name:**<br>**Position:**<br>**Phone:**<br>**Email:**
**Potential key informant 2 (Gr2/CNS midwife)**	**Name:**<br>**Position:**<br>**Phone:**<br>**Email:**
**Potential key informant 3 (Medical Practitioner)**	**Name:**<br>**Position:**<br>**Phone:**<br>**Email:**

Hospital address:

DO NOT COPY

Checklist

Please check that the following are attached:

- ☐ Completed **hospital survey**
- ☐ **Documentation** used in the provision of postnatal care including: care maps/pathways; progress notes; medication charts; observation charts; postnatal protocols; postnatal guidelines; postnatal standards or any other documentation as applicable.
- ☐ **Consumer information** on the postnatal period: leaflets/brochures
- ☐ The names and contact details of **three key informants**

Please return the above to Mother & Child Health Research, in the Reply Paid envelope that accompanied the survey. Should you have misplaced the envelope please contact Jo Rayner on 8341 8533.
Our mailing address is:

MCHR, La Trobe University
251 Faraday St,
CARLTON, Vic. 3053

Once again our thanks for your involvement in PinC Private.

DO
