# Supplementary material for: A statewide review of postnatal care in private hospitals in Victoria, Australia
Source: BMC Pregnancy Childbirth. 2010 May 28;10:26. doi: 10.1186/1471-2393-10-26 (PMC2891607; doi:10.1186/1471-2393-10-26)
Supplement: Additional file 2 — Glossary of Terms. A list of descriptive terms used in the paper which may be specific to Australia. [file 1471-2393-10-26-S2.DOC]

**Additional File 2: Glossary of terms.**

*Breastfeeding day stay services*

A lactation support service where women can be admitted for several hours to receive information and assistance with breastfeeding.

*Care maps*

Also called clinical pathways and are used to achieve continuity of care across various care providers and to ensure all aspects of care are covered.

*Casual bank*

Hospital employed nurses and midwives who provide care on a casual basis.

*Division 2 nurse*

Division 2 nurses, previously called enrolled nurses, provide care under the direction of Division 1 nurses or midwives, who are licensed to practice nursing in the field/s. in which they are registered (Nurses Board of Victoria 2001).

*Enrolled nurse*

See Division 2 nurse above.

*Hotel care*

The provision of some of the postnatal stay in a hotel, which is usually confined to low risk women, and covered by private health insurance.

*Mothercraft nurse*

Mothercraft nurses are trained to care for children from birth to five years old, how this qualification is now redundant.

*Well-baby nursery*

A nursery where mothers can leave their infants to be cared for by hospital staff during their postnatal stay, in particular for their convenience during rest times or overnight.
